# MRSNet: Multi-Resolution Scale Feature Fusion-Based Universal Density Counting Network

**DOI:** 10.3390/s24185974

**Published:** 2024-09-14

**Authors:** Yi Zhang, Wei Song, Mingyue Shao, Xiangchun Liu

**Affiliations:** 1 School of Information and Engineering, Minzu University of China, Beijing 100081, China; 22302002@muc.edu.cn (Y.Z.); smy19990312@gmail.com (M.S.); lxc@muc.edu.cn (X.L.); 2 Language Information Security Research Center, Institute of National Security MUC, Minzu University of China, Beijing 100081, China; 3 National Language Resource Monitoring and Research Center of Minority Languages, Minzu University of China, Beijing 100081, China; 4 Key Laboratory of Ethnic Language Intelligent Analysis and Security Governance of MOE, Minzu University of China, Beijing 100081, China

**Keywords:** crowd counting, CNN network, dense target counting, density map

## Abstract

This study focuses on the problem of dense object counting. In dense scenes, variations in object scales and uneven distributions greatly hinder counting accuracy. The current methods, whether CNNs with fixed convolutional kernel sizes or Transformers with fixed attention sizes, struggle to handle such variability effectively. Lower-resolution features are more sensitive to larger objects closer to the camera, while higher-resolution features are more efficient for smaller objects further away. Thus, preserving features that carry the most relevant information at each scale is crucial for improving counting precision. Motivated by this, we propose a multi-resolution scale feature fusion-based universal density counting network (MRSNet). It utilizes independent modules to process high- and low-resolution features, adaptively adjusts receptive field sizes, and incorporates dynamic sparse attention mechanisms to optimize feature information at each resolution, by integrating optimal features across multiple scales into density maps for counting evaluation. Our proposed network effectively mitigates issues caused by large variations in object scales, thereby enhancing counting accuracy. Furthermore, extensive quantitative analyses on six public datasets demonstrate the algorithm’s strong generalization ability in handling diverse object scale variations.

## 1. Introduction

The task of dense object counting aims to quantify the number of objects in complex scenes. With advancements in deep learning and computer vision technologies, the demand for intelligent counting tasks has greatly driven research in dense object counting. Furthermore, significant improvements in hardware performance have expanded the application scenarios, such as counting crowds [1,2,3,4] to monitor real-time crowd density, analyze trends in crowd gathering and movement, ensure safety measures in places like stations and scenic areas, and prevent stampedes—a crucial aspect of public safety management. Counting vehicles [5,6] helps reduce traffic congestion and effectively manages traffic flow. Counting cells aids in medical diagnostics, reducing errors associated with manual counting. Counting animals [7,8,9] is essential for monitoring wildlife populations and scientifically managing livestock in agriculture. Counting crops [10] helps in estimating crop yields and assessing planting success rates scientifically.

Deep-learning-based dense object counting algorithms have significantly reduced the laboriousness of manual counting while minimizing errors, thus offering considerable practical application value. However, counting in dense scenes still faces numerous challenges. Highly crowded scenes present irregular object distributions and uneven object scales. Traditional convolutional neural networks struggle with fixed-size kernels for feature extraction, limiting counting performance. The current approaches primarily focus on two solutions: learning scale factors and multi-scale fusion. Scale factor learning estimates appropriate scales from image features to adjust block sizes for density map prediction, necessitating additional scale learning during training. Multi-scale fusion methods further divide into feature fusion [11,12,13] and density map fusion [14,15,16]. Multi-scale feature fusion, represented by attention mechanisms, combines shallow and deep features for counting tasks, while multi-scale density map fusion integrates hierarchical counting results to improve counting accuracy. These methods have shown success with multi-scale objects but still struggle with feature scale variations in highly crowded scenes.

In the same scenes, higher-resolution information typically offers stronger representation capabilities. Existing methods often preserve high-resolution information through networks that sequentially pass inputs through sub-networks of decreasing resolution, using symmetric processes to restore high-resolution features [17]. Alternatively, they utilize residual structures, transpose convolutions, and other operations to reverse-generate high-resolution information [18]. Previous research [19,20] indicates that different resolutions exhibit varying degrees of scale bias. Single-resolution features are limited to representing target features within specific scale ranges, failing to apply universally across all scales.

Building on this research foundation, we propose a more efficient architectural approach based on a multi-resolution network. It integrates multi-scale selection feature modules and a dynamic sparse attention mechanism to accommodate large-scale variations in target objects. Unlike existing processes that restore resolution from low to high, our method parallelly connects sub-networks of different resolutions from high to low, gradually increasing the number of sub-network layers to repetitively fuse multi-scale information. This approach effectively preserves high-resolution features, avoiding feature information loss and enhancing the accuracy of density map predictions.

Simultaneously, our multi-scale selection feature module decomposes each feature into high- and low-resolution components before each stage of feature fusion, enabling independent processing. It adapts the receptive field size adaptively according to input scale information, guiding softmax attention to fuse multiple branches of different scales. Different resolutions receive varying attention in the fusion layer, optimizing neuron effective receptive field sizes. Through stacked multi-scale selection modules, the network gains the ability to adaptively adjust receptive field sizes based on inputs, capturing target objects across different resolutions.

Complementarily, the dynamic sparse attention mechanism removes the least relevant key–value pairs in coarse-grained areas, efficiently locating valuable key–value pairs and optimizing feature information at each resolution. This process combines higher-resolution features with lower-resolution features to ensure the effective capture of objects within each scale range, presenting a straightforward and efficient implementation of dual-route attention.

The problem of dense object counting is of significant importance in the field of computer vision, particularly in scenarios involving crowded and densely packed environments. However, the existing methods face various challenges and limitations in addressing this problem, which severely restrict the accuracy of counting.

Firstly, objects in dense scenes often exhibit substantial scale variations, posing a significant challenge to counting algorithms. Current convolutional neural network (CNN) methods typically use fixed-size convolutional kernels, which results in lower efficiency when handling objects of varying scales. Similarly, Transformer-based methods rely on fixed-size attention mechanisms, which also struggle to accommodate diverse object scales. These fixed feature extraction and attention mechanisms frequently fail to effectively manage the scale variations in dense scenes, leading to a notable decrease in counting accuracy.

Secondly, low-resolution features are generally more sensitive to large objects close to the camera, while high-resolution features are more efficient for small-scale objects that are farther away. Consequently, a critical issue with the current methods is how to optimally retain feature information at different resolutions to enhance overall counting accuracy.

To address these challenges, we propose MRSNet. The motivation for our research arises from a thorough analysis of the limitations of existing methods. By introducing separate module channels to handle high-resolution and low-resolution features, we can adaptively adjust the receptive field size and integrate a dynamic sparse attention mechanism to optimize feature information at each resolution. This approach not only effectively alleviates issues related to object scale variations but also integrates optimal features across different scales to produce accurate density maps for counting evaluation.

Our main contribution includes proposing the MRSNet, which distinguishes between high- and low-resolution features, adapts receptive field sizes dynamically, integrates a dynamic sparse attention mechanism, optimizes feature information at each resolution, and ultimately fuses optimal features across multiple scales to address scale variations and improve counting accuracy. We achieved promising results on various public counting tasks, validating the effectiveness of our model. The primary research scenario is crowd counting, while vehicle and plant counting serve to expand the scope of experimental data and demonstrate the versatility of the model. These supplementary experimental data facilitate the verification of the method’s broad applicability and enhance the model’s performance in diverse dense scenes.

## 2. Related Works

Currently, mainstream methods for dense object counting primarily focus on crowd counting. The powerful feature extraction capability of convolutional neural networks in deep learning has accelerated the development of research into automatically extracting features and training end-to-end networks for individual counting.

Based on deep learning technology, these approaches can roughly be categorized into three types: detection-based methods, regression-based methods, and density map estimation methods.

### 2.1. Detection-Based Methods

Early research methods were mostly based on detection frameworks, using sliding windows to detect target information in scenes for counting [21,22]. Detection-based methods heavily rely on feature extraction, which can be broadly categorized into global and local approaches. Global detection methods [23,24,25,26] are typically traditional pedestrian detection methods that train classifiers using features extracted from the entire body. They perform well in sparse crowd scenes but show noticeable performance degradation in high-density crowds. Some studies have attempted to address this issue by employing part-based detection methods [27,28,29], which involve constructing enhanced classifiers for specific body parts to estimate the number of individuals in designated areas [30]. Zhao et al. [31] utilized shape learning to model humans using 3D shapes composed of ellipsoids, while Leibe et al. [25] combined top-down probabilistic segmentation of local and global cues, further expanding the approach.

As crowd density increases or encounters more complex scenes, severe instance overlap becomes a problem. This can lead to decreased accuracy in predicted bounding boxes. Additionally, highly overlapped targets may have very similar features, making it difficult for detectors to generate distinct predictions for each instance. Therefore, the accuracy of detection-based crowd counting algorithms significantly decreases, resulting in inaccurate counting results [27,32,33]. Hence, detection-based methods are only suitable for low-density crowds, where they have shown better performance in sparse target scenarios. However, when counting targets in high-density scenes, detectors struggle to achieve satisfactory performance due to occlusions, scale variations, and other factors. Depending solely on detection methods fails to meet the demand for high-accuracy counting.

### 2.2. Regression-Based Methods

Regression-based counting methods avoid reliance on detectors by leveraging the mapping between image features and individual counts, demonstrating the effectiveness of the paradigm of low-level feature extraction and regression modeling. The concept of regression-based crowd counting was initially introduced by Davies et al. [34], who proposed regressing crowd counts from raw features such as total edge counts and foreground area. Subsequent regression-based methods have followed similar processing steps: initially encoding global features like textures, gradients, or edges, then employing various regression strategies such as linear regression [35], piecewise linear regression [36], or Gaussian process regression [37] to learn mappings from low-level features to counts of target objects. Developing regression models in this manner establishes mappings between actual counts and estimated counts.

While early regression methods effectively addressed occlusion and clutter issues, many of them regressed on global information, thereby overlooking crucial spatial details.

### 2.3. Density Map Estimation Methods

Further research has led to a significant increase in interest in the concept of density maps, as proposed by Lempitsky et al. [38]. Density map estimation methods entail the utilization of convolutional neural network models for the prediction of crowd scene density maps, which reflect the number of individuals with spatial location information. The model is trained to map images to density maps for the purpose of counting, thereby circumventing the challenges inherent in detecting and locating individual object instances. The integral over any region in the density map provides the count of objects within that area. Rodriguez et al. [39] demonstrated that the use of density maps for counting purposes markedly improves the accuracy of such operations.

Integral-based approaches encounter challenges in addressing large-scale and density variations, whereas block-based methods integrate more local image information and demonstrate less sensitivity to scale and density alterations. In a related study, Pham et al. [40] proposed a method for learning nonlinear mappings between patches and density maps. This approach employed random forest regression for density estimation from multiple image blocks. The images were segmented into blocks, and random forests were employed to classify features, resulting in a notable enhancement in model performance. Wang et al. [41] introduced an innovative modification to the model architecture, namely a rapid density estimation method based on subspace learning. This approach avoids the direct learning of mappings between dense features and their corresponding density maps, instead focusing on the embedding of each subspace formed by image blocks.

In essence, these methodologies capitalize on the interrelationships between images and their associated density maps within the domain of feature space. Density maps not only reflect the spatial distribution of dense targets but also enhance the accuracy of counting, thereby making density map estimation a rapidly developing area of research. While detection-based counting is optimal in sparse regions, regression-based counting is more effective in dense areas. The processing of multi-scale distributions of targets in complex scenes represents a significant challenge for counting. Due to the inherent variability in object scale resulting from disparate imaging perspectives, objects situated in closer proximity to the camera appear larger with a greater number of pixels, whereas those located at a greater distance possess a smaller number of pixels. Consequently, the estimation of density is affected by the uneven distribution of pixels belonging to the same object. We put forth a density-map-based parallel structure counting framework that discerns features at the pixel level.

## 3. Methods

**Figure 1:**
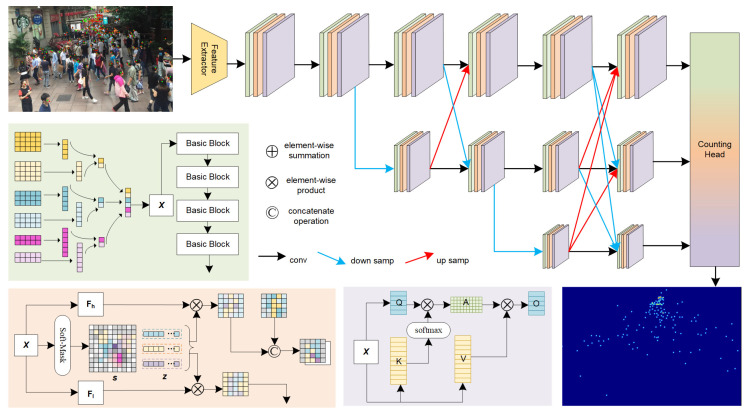
This is a MRSNet architecture. The primary network structure is illustrated in the figure, comprising several key components: a basic unit module (in green) consisting of a series of basic convolutional blocks along with upsampling and downsampling operations; a multi-scale feature selection module (in orange); and a dynamic sparse attention module (in purple). Finally, all the information is consolidated into the counting head, where density map generation is achieved through Gaussian kernel integration regression (depicted by the gradient transition from green to orange to purple).

This section presents a detailed account of the MRSNet framework. It elucidates the fusion of multi-scale selected feature modules and dynamic sparse attention mechanisms [42] within the context of a multi-resolution network. In order to address the considerable range of scale variations observed in the target object, the fundamental diagrammatic representation is illustrated in Figure 1.

### 3.1. Multi-Resolution Network

In deep learning algorithms, it is common to employ multi-resolution features to capture multi-scale objects. Lower-resolution features are more sensitive to larger objects closer to the camera lens, while higher-resolution features are more efficient for smaller-scale objects farther away. Therefore, establishing a multi-resolution feature representation is crucial.

The network is constructed in multiple stages, where each stage progressively lowers the resolution of sub-networks as new stages are parallelly connected. The resolution of the sub-networks includes all resolutions from the previous stage plus a new lower resolution. Information exchange between parallel sub-networks is repeated to achieve integration of information feature maps across different resolutions. This study primarily utilizes the optimized structure following HRNet-W48 [43] as the backbone, composed of three stages of parallel sub-networks. Their resolutions gradually decrease by half, while their widths (number of channels) correspondingly double. Each stage’s design consists of basic convolution units, multi-scale feature selection modules, and dynamic sparse attention.

**Figure 2:**
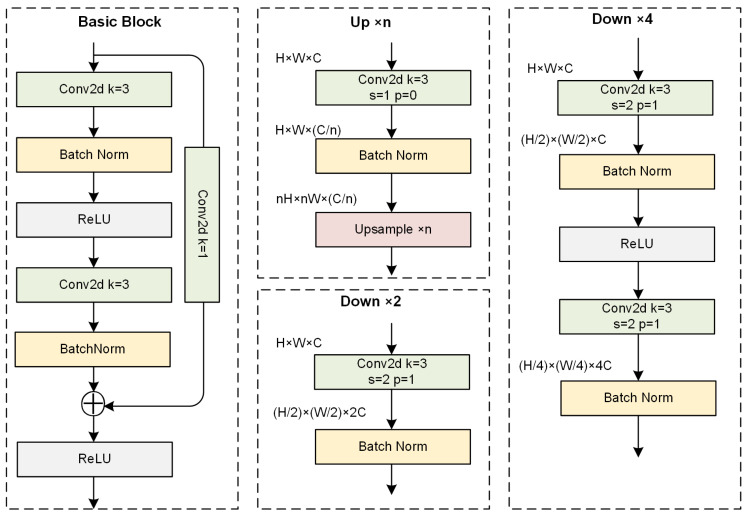
Detailed structure diagram of the basic module. BasicBlock specifies the specific structure for feature extraction. Up denotes the operation for upsampling by a factor of n. Down consists of structures for downsampling by factors of 2 and 4, respectively.

In Figure 1, the basic unit module (green) consists of a sequence of four basic convolutional blocks, combined with upsampling (Up) and downsampling (Down) operations. The detailed structure is illustrated in Figure 2. Each BasicBlock is composed of a residual structure with two 3 × 3 convolutions and one 1 × 1 convolution. This module is an essential component of the three parallel sub-network stages and is crucial due to the role of upsampling and downsampling operations in adjusting the spatial resolution of feature maps. For upsampling, we use a simple adjacent sampling followed by 1 × 1 convolution and batch normalization layers to align the channel dimensions. For downsampling, various convolution kernel sizes are applied. The output of each scale branch is obtained by fusing the outputs from all branches. For instance, the output of the 2× downsampling branch is obtained by adding the upsampled outputs of the 2× and 4× downsampling branches, followed by a ReLU activation to produce the final fused output for the 2× downsampling branch. Other branches follow a similar approach.

Assuming an RGB image $I\in R^{3\times H\times W}$, where $\Phi_{\theta a}$ denotes the backbone network with $\theta_{a}$ as its parameters. Then, N + 1 multi-scale features can be represented as ${\left\{R_{j}\right\}}_{j=0}^{N}=\Phi_{\theta_{a}}\left(I\right)$, where the spatial resolution at the *j*-th level is $\left(h_{j},w_{j}\right)=\left(\frac{H}{2^{j+2}},\frac{w}{2^{j+2}}\right)$. Image features undergo information fusion through a MRSNet, and the features output from the final stage’s sub-network are converted into a density map through a counting regression head.

### 3.2. Multi-Scale Feature Selection Module

Based on prior research, efficient features can only be generated within a certain scale range at each resolution. Therefore, the primary motivation of our multi-scale feature selection module is to preserve high-resolution features from each stage to the fullest extent possible, progressively integrating lower-resolution features. When the input streams are $R_{j-1}$ and $R_{j}$, and the output streams are $O_{j-1}$ and ${\bar{R}}_{j-1}$, the information flow through the multi-resolution selection module can be represented as follows:

(1) $$O_{j-1}=C\left\{R_{j-1},A_{h}⨀{\;C}_{\theta_{c}}{\bar{R}}_{j}\right\}$$

(2) $${\bar{R}}_{j-1}=C\left\{R_{j-1},A_{l}⨀\;U_{\theta_{u}}\left({\bar{R}}_{j}\right)+A_{h}\;⨀C_{\theta_{c}}\left({\bar{R}}_{j}\right)\right\}$$

where, C denotes feature concatenation, with $A\in R^{2\times h_{j}\times w_{j}},j=1,\;2,\;...,\;N$ represents a dual-channel attention mechanism, split into a high-resolution channel $F_{h}$ and a low-resolution channel $F_{l}$ along the channel dimension. ⊙ denotes the Hadamard product.

In the current stage, high-resolution features selected from the previous stage are inherited, ensuring that $O_{j-1}$ maintains its ability for fine-grained prediction after fusion with the current stage features. Operations at each stage are similar, culminating in the aggregation of all objects at the highest resolution for final counting operations.

**Figure 3:**
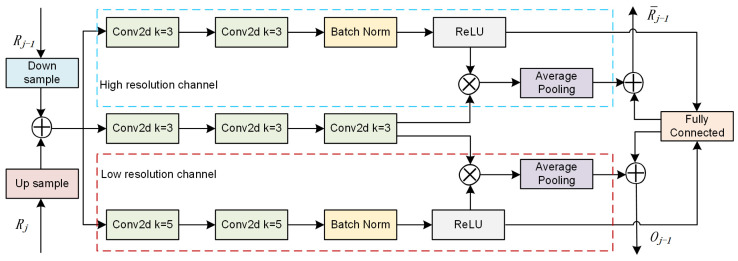
Detailed structure diagram of the multi-scale feature selection module. When the input streams are $R_{j-1}$ and $R_{j}$, and the output streams are $O_{j-1}$ and ${\bar{R}}_{j-1}$, this module segregates features into high-resolution and low-resolution channels for separate processing.

Our proposed multi-scale feature selection module employs gating mechanisms to process high-resolution and low-resolution features separately, adaptively adjusting receptive field sizes. This allows multiple branches carrying information from different scales to converge into the information flow of neurons in the next layer. The module structure, as depicted in Figure 3, consists of three components: high-resolution feature processing block, low-resolution feature processing block, and mask generation block. Each component includes convolutional layers with varying depths and batch normalization, along with ReLU activation functions. The high-resolution feature processing block extracts features above a threshold and preserves them, while the low-resolution feature processing block forwards features below the threshold to the subsequent stage of the network.

For any given feature map $X\in R^{H^{\prime }\times W^{\prime }\times C^{\prime }}$, resolution selection is initially performed, dividing the features into $\tilde{F}:X\to \tilde{U}\in R^{H\times W\times C}$ and $\hat{F}:X\to \hat{U}\in R^{H\times W\times C}$, with kernel sizes set to 3 and 5, respectively. Both $\tilde{F}$ and $\hat{F}$ consist of grouped convolutions, batch normalization, and activation functions.

Element-wise fusion of information flow from the previous stage yields the following result: $U=\tilde{U}+\hat{U}$, using gating mechanisms to adjust information flow.

Subsequently, we embed the global information by simply employing global average pooling (ap) to generate channel-wise statistics, such as $s\in R^{C}$, Specifically, the *c*-th element of *s* is computed by reducing U along the spatial dimensions *H* × *W*.

(3) $$s_{c}=F_{ap}\left(U_{c}\right)=\frac{1}{H\times W}\sum_{i=1}^{H}\sum_{j=1}^{W}U_{c}\left(i,j\right)$$

Additionally, a dense feature $z\in R^{d\times 1}$ has been created to facilitate precise and adaptive selection. This is achieved through a simple fully connected (fc) layer, reducing dimensions for improved efficiency.

(4) $$z=F_{fc}\left(s\right)=\delta \left(B\left(Ws\right)\right)$$

Additionally, where *δ* denotes the ReLU function, *B* represents batch normalization, and $W\in R^{d\times C}$.

### 3.3. Dynamic Sparse Attention Mechanism

To efficiently locate valuable key–value pairs for global reference, the integration of a dynamic sparse attention mechanism enables the model to further focus on the most valuable high-resolution features at each stage. It filters out the least relevant key–value pairs at the low-resolution level rather than directly filtering them at the high-resolution level.

In the MRSNet, the dynamic sparse attention mechanism is integrated to achieve more flexible computation allocation and content awareness through two-level routing. Specifically, the objective is for each query to focus on the most relevant parts of the key–value pairs.

Given the feature map $X\in R^{H\times W\times C}$, it is initially divided into n×n non-overlapping regions, each containing feature vectors representing $HW/n^{2}$. Linear projections are then used to derive tensors for queries, keys, and values. The dynamic sparse attention mechanism operates in a query-adaptive manner. Specifically, for queries, it first filters out irrelevant key–value pairs at the coarse region level and then applies fine-grained token-to-token attention to the union of the remaining candidate regions (i.e., routing regions).

**Figure 4:**
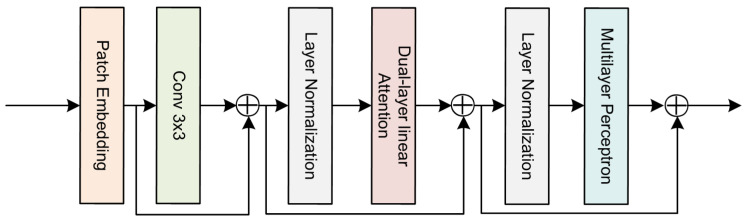
Detailed Structure Diagram of Dynamic Sparse Attention Mechanism. Comprising convolutional layers, normalization layers, dual-layer linear attention, and multi-layer perceptrons.

Relative position information is implicitly encoded using a 3 × 3 depth convolution. Layer normalization, a dual-layer linear attention module, and a multi-layer perceptron module are successively applied to the modeling of cross-location relationships and location embeddings, as depicted in Figure 4. Using dual-layer linear attention as a foundational component, the process operates as follows: in the first layer, the input features undergo an initial linear transformation, mapping them to an intermediate space. This step extracts the principal information from the features while reducing dimensionality, thereby enhancing computational efficiency; in the second layer, the intermediate features are subsequently subjected to a second linear transformation to generate the final attention weights. This enables the model to perform feature fusion in a higher-dimensional space, thus improving its ability to capture complex patterns. By employing these two sequential linear transformations, the model achieves feature extraction and fusion at multiple levels, which enhances its performance and flexibility. This design is particularly well-suited for managing the multi-scale and multi-level information produced by our network’s parallel architecture. It facilitates content-aware processing of the most relevant keys/values for each query, thereby enabling more precise feature selection and improved counting performance.

Using cross-channel softmax to adaptively select different information spatial scales, guided by dense feature descriptors *z*. Specifically, applying the softmax operator along the channel dimension:

(5) $$a_{c}=\frac{e^{A_{c}z}}{e^{A_{c}z}+e^{B_{c}z}},\;b_{c}=\frac{e^{B_{c}z}}{e^{A_{c}z}+e^{B_{c}z}}$$

where $A,B\in R^{C\times d}$, *a* and *b*, respectively, represent the softmax operations applied to $\tilde{U}$ and $\hat{U}$. $A_{c}\in R^{1\times d}$ is the *c*-th row of *A*, and $a_{c}$ is the *c*-th element of *a*. Similarly, $B_{c}$ and $b_{c}$ follow the same notation. In the case of two branches, matrix *B* is redundant because $a_{c}$ + $b_{c}$ = 1. The final feature map is obtained by weighting attention across different scales.

(6) $$V_{c}=a_{c}\cdot \tilde{U}+b_{c}\cdot \hat{U},\;a_{c}+b_{c}=1$$

Finally, we introduce the counting head $E_{\theta e}$, where $\theta_{e}$ represents its parameters. These parameters are solely trained by the final output branch to aggregate all objects at the highest resolution for the ultimate prediction. The overall loss function of the network is expressed as follows:

(7) $$l=\sum_{j=1}^{N}\alpha_{j}l_{j}=\sum_{j=1}^{N}\alpha_{j}L\left({E_{\theta e}(a}_{c}\cdot \tilde{U})⨀{\hat{C}}_{i}^{gt},{E_{\theta e}(b}_{c}\cdot \hat{U}){⨀C}_{i}^{pred}\right)$$

where ${\hat{C}}_{i}^{gt}$ denotes the ground truth and $C_{i}^{pred}$ denotes the predicted value, $\alpha_{j}$ represents the weights for each resolution, empirically set as $\alpha_{j}=1/2^{j}$. L denotes the Euclidean distance.

## 4. Experimentation

In order to validate the effectiveness of the MRSNet framework for dense object counting, we conducted a series of experiments and performed detailed analysis of the experimental results. Visualizing the outcomes further enhances the clarity and objectivity of the algorithmic results, ultimately substantiating the efficacy of the model.

### 4.1. Datasets

We selected six commonly used datasets that include dense object counting. For different targets, we comprehensively evaluated the algorithms as follows: population counting datasets include SHHA [44], SHHB [44], UCF-QNRF [45], NWPU-Crowd [46]; vehicle counting datasets include TRANCOS [47]; and plant counting includes MTC [10].

ShanghaiTech is a classic public dataset suitable for dense population counting. It is divided into SHHA and SHHB based on different density distributions. Part A consists of 482 images collected from the internet, with 300 images for training and 182 for testing. Part B includes 400 images captured in the urban streets of Shanghai, with 400 for training and 316 for testing. The scale variations and viewpoint distortions in this dataset present new challenges and opportunities for many CNN-based counting network designs.

UCF-QNRF, released by the University of Florida, is widely used for crowd counting tasks. It comprises 1535 images with 1201 images for training and 334 for testing, with annotations ranging from 49 to 12,865 per image. Compared with other datasets, UCF-QNRF contains widely annotated human bodies across multiple scenes, viewpoints, lighting conditions, and density changes, and the diverse research scenarios better test the generalization performance of the model.

NWPU-Crowd, published by Northwestern Polytechnical University, contains 5109 images covering the nighttime environment and various lighting scenes, which further enriches the sample diversity with 2,133,238 annotated instances. In addition to its large data volume, this dataset offers advantages such as negative samples, fairness in evaluation, high resolution, and significant appearance variations compared to previous real-world datasets.

TRANCOS is the first system for vehicle counting in congested traffic images, featuring 1244 images from different congested traffic scenes captured by surveillance cameras with annotations for 46,796 vehicles. This dataset is commonly used to assess the generalization capabilities of dense object counting methods.

MTC consists of 361 high-resolution images of corn tassels in outdoor fields. Unlike objects with similar physical sizes, corn tassels exhibit heterogeneity in physical dimensions and undergo self-change over time, making them suitable for evaluating the robustness of models designed for size variations of objects.

### 4.2. Metrics

To assess the qualitative metrics of dense object counting algorithms, we evaluated using mean absolute error (MAE) and mean square error (MSE).

MAE is the most commonly used evaluation metric in object counting tasks, indicating the Manhattan distance between the actual and predicted counts in an image. It measures the sum of the absolute differences between the predicted and true values, but it is insensitive to outliers.

(8) $$MAE=\frac{1}{N}\sum_{i=1}^{N}\left|C_{i}^{pred}-{\hat{C}}_{i}^{gt}\right|$$

MSE assesses the accuracy of prediction models or estimation methods by calculating the average of the squares of the differences between predicted and actual values. It is sensitive to outliers and can be used to evaluate their impact. The definitions of these evaluation metrics are as follows:

(9) $$MSE=\frac{1}{N}\sum_{i=1}^{N}{\left(C_{i}^{pred}-{\hat{C}}_{i}^{gt}\right)}^{2}$$

where, N represents the number of objects, ${\hat{C}}_{i}^{gt}$ is the ground truth count for the *i*-th query image, and $C_{i}^{pred}$ is the predicted count for the *i*-th query image.

### 4.3. Environment of the Experiment

The training and testing for this experiment were conducted on a server running Ubuntu 20.04.1 operating system, equipped with three NVIDIA GeForce RTX 2080 GPUs, each with 8 GB of memory. The environment utilized CUDA version 11.4 and Python 3.9. Training employed the Adam optimizer over 800 epochs, incorporating a linear warm-up strategy for the initial 10 epochs, followed by cosine decay. The learning rate was gradually increased from 0 to 10^−5^.

### 4.4. Analysis of the Results of Dense Crowd Counting

Dense crowd counting is a critical subtask within dense counting. We evaluated our model on four challenging crowd counting datasets: SHHA, SHHB, UCF-QNRF, and NWPU-Crowd.

Across all datasets, MRSNet demonstrated strong performance, outperforming existing results on some datasets. Table 1 shows the comparison results with other algorithms. Our method demonstrates superior performance on the UCF-QNRF and NWPU datasets. On the UCF-QNRF test set, our method achieves an MSE of 130.4 and an MAE of 78.5, significantly outperforming existing methods. For the NWPU dataset, our method reduces the MAE and MSE to 69.3 and 319.7, respectively, surpassing all comparative algorithms and currently holding the top position. Additionally, on the SHHA and SHHB datasets, the MAE of our method deviates by only 1.5 and 0.1 from the best reported results, respectively. These findings indicate that our method exhibits exceptional performance in crowd counting across various datasets.

**Figure 5:**
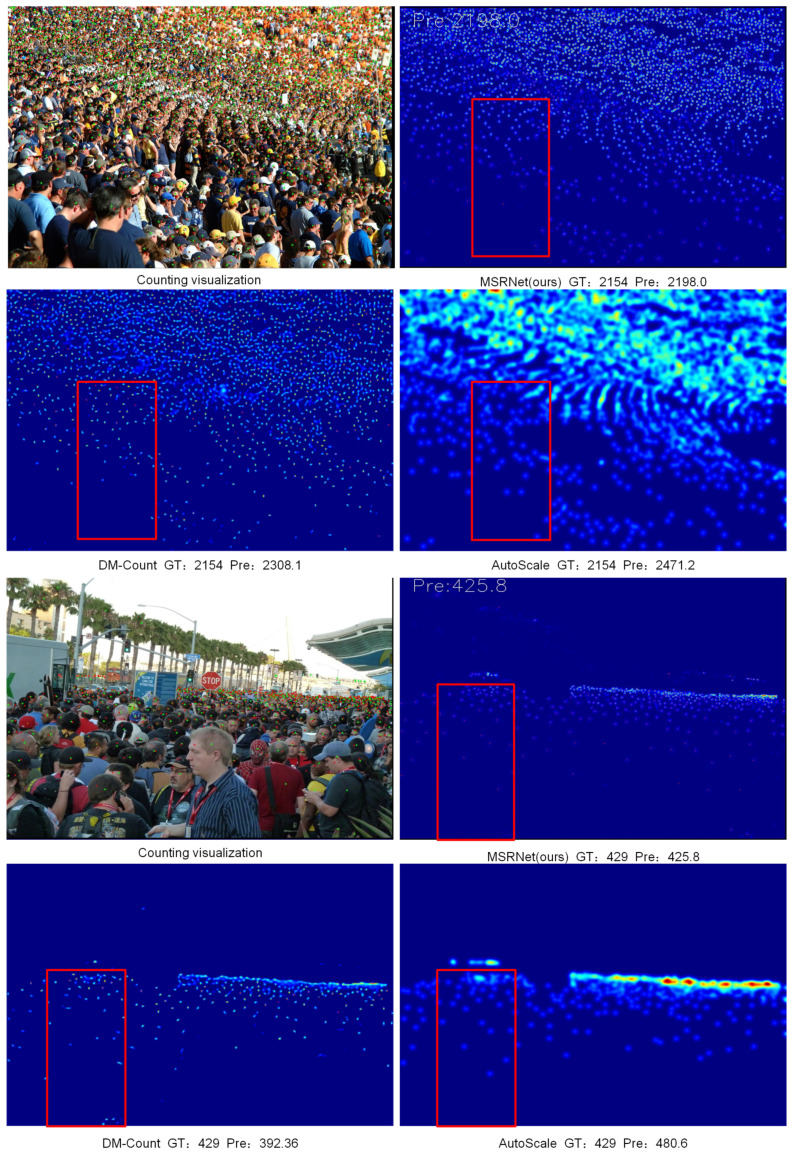
Visualize the comparison. Visualization results of different algorithms facing drastic scale changes in dense crowds. The red box is the area where the crowd density changes sharply, and the higher the brightness, the greater the density.

Figure 5 demonstrates the superiority of our model in handling scale variations within dense crowds. The first image depicts a densely packed audience where individuals closer to the camera are larger, occupying more pixels and having higher resolution, whereas those farther away are smaller, with some barely comprising a few pixels. Particularly noticeable in the bottom-left corner, we handle these larger, closer targets with a high-resolution module, while smaller, distant targets are processed using a low-resolution module. Feature extraction progresses hierarchically from near to far, aiming to preserve high-resolution features as much as possible. Our predictions closely approximate ground truth, achieving an error of 44 with a count of 2198.0. In contrast, competing algorithms struggle to grasp similar target scale variations, often misinterpreting larger, closer targets as overlapping smaller dense objects, leading to increased counting errors. The second image similarly illustrates our model’s adeptness at handling scale variations in dense scenes, predicting 425.8 individuals compared to a ground truth of 429, with a discrepancy of only 3.2. In contrast, competing algorithms show discrepancies of 36.64 and 51.6, respectively. These results validate the robustness and effectiveness of our model in addressing scale variations of targets in dense scenarios.

### 4.5. More Dense Counting Application Results

In this section, we present additional experimental data challenging our dense counting tasks applied to diverse scenarios. Table 2 illustrates the outstanding performance of our network MRSNet on the vehicle counting dataset TRANCOS, the outdoor corn tassel dataset MTC, and the urban tree dataset, demonstrating the versatility and strong generalization capability of our approach.

**Figure 6:**
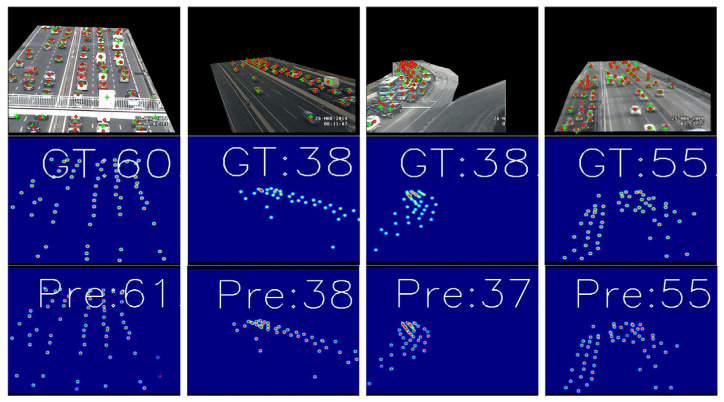
TRANCOS visualizes the results. The first row shows the count visualization on the original image, the second row shows the true value of the density map, and the third row shows the predicted value of our network density map.

In terms of vehicle datasets, our model achieved a decrease in MAE to 1.7 and MSE to 3.0 for vehicle detection tasks. Figure 6 illustrates several representative samples. The first column from the left shows instances with occlusion and significant scale variations, where vehicles appear smaller in the distance and larger when closer to the camera, with an error of 1 between ground truth and predictions. Columns two through four depict real-world scenarios with uneven distributions of targets. Our model effectively mitigates these challenges, capturing each target more accurately and minimizing prediction errors.

**Figure 7:**
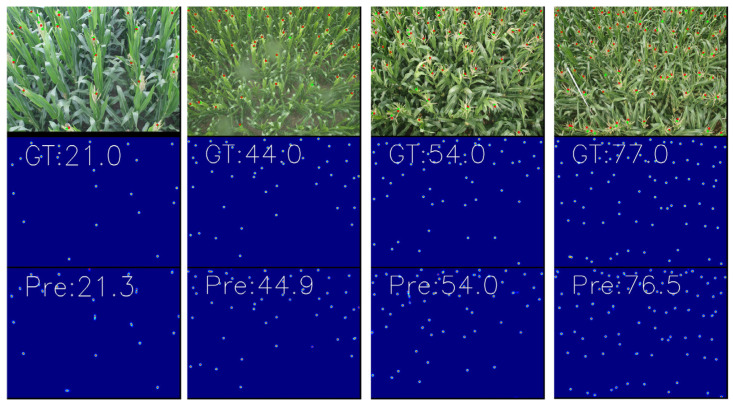
MTC Visualization Results. The first row shows the count visualization on the original image, the second row shows the true value of the density map, and the third row shows the predicted value of our network density map.

In plant counting, our model has surpassed other state-of-the-art (SOTA) models, demonstrating significant improvements on the MTC dataset with MAE reduced to 2.6 and MSE to 3.7. Figure 7 showcases several representative samples. Starting from the left in the first column, it depicts scenarios with considerable scale variations where corn tassels are larger closer to the camera and smaller in the distance. This effectively demonstrates our model’s capability to handle significant scale variations, achieving an error of 0.3. In the second column, even with rainwater stains on the camera lens, accurate identification is maintained with an error of 0.9. The third and fourth columns illustrate dense corn tassels and complex foliage in the same frame, scenarios that typically challenge human detection. Our model minimizes errors to within 0.5, proving its robustness and reliability.

### 4.6. Ablation Experiment

In this section, we conduct ablation experiments on the publicly available SHHB dataset to assess the impact of each module on the network performance. We use the original unmodified HRNet as the baseline network and report quantitative results in Table 3. Subsequently, we evaluate the integrated effects of two modules at different positions within the network to investigate their optimal contributions to overall network performance.

The independent impacts of the two modules were validated separately. It can be observed that when embedding only the multi-scale feature selection module, the test set MAE decreased by 4.1 and RMSE by 4.6 compared to the baseline. When incorporating only the dynamic sparse attention mechanism, the test set MAE decreased by 2.2 and RMSE by 3.2 compared to the baseline. These results indicate significant enhancements in counting performance due to both modules, with the multi-scale feature selection module showing more pronounced effects. The lowest error metrics on the validation and test sets were achieved when both Block1 and Block2 modules were present, with reductions of 5.6 and 6.9 on the test set, respectively. This demonstrates complementary contributions when used together, significantly enhancing the overall counting accuracy of the model.

**Figure 8:**
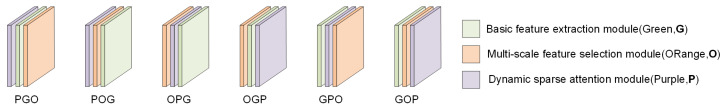
Diagram of different arrangements and combinations of modules. Right is the legend, green (G) represents the main network for basic feature extraction, orange (O) represents the multi-scale feature selection module, and purple (P) represents dynamic sparse attention.

We conducted validation experiments to assess the effects of arranging the two modules at different positions, as depicted in Figure 8. In the diagram, green represents the base feature extraction structure, orange represents the multi-scale feature selection module, and purple represents the dynamic sparse attention mechanism. Depending on the different positions within the backbone network and the order in which the two modules are connected, there are a total of six different arrangement combinations.

We found that placing this mechanism at different levels affects the network performance to varying degrees. According to the experimental results in Table 4, sequentially connecting the multi-scale feature selection module and dynamic sparse attention mechanism after the backbone feature extraction significantly enhances the overall performance of the counting network. This indicates that the sequence of combining these modules plays a crucial role in optimizing the network structure.

## 5. Conclusions

This paper proposes a parallel structure counting framework based on density map counting: multi-resolution scale feature fusion network, addressing the challenge of multi-scale variations in dense object counting. Our method, MRSNet, utilizes a multi-scale feature selection module to differentiate high-resolution features and low-resolution features processed independently through separate channels. It adaptively adjusts the receptive field size and integrates a dynamic sparse attention mechanism to optimize feature information at each resolution. Ultimately, it fuses optimal features from multiple scales to cope with scale variations. The experimental results demonstrate that our approach performs well in traditional crowd counting scenarios as well as in practical applications such as vehicle and crop counting. The model has a certain dependence on data resources and hardware resources in the actual deployment. In the future, we will consider further optimizing the model to break through the limitations and challenges in practical applications.

## Figures and Tables

**Table 1:** Comparison experimental results of SHHA, SHHB, UCF-QNRF, and NWPU-Crowd. Quantitative results of different methods on the test set of four publicly available datasets. Comparison of experimental results first (red), indicators second (blue).

Methods	SHHA		SHHB		UCF-QNRF		NWPU	
	MAE	MSE	MAE	MSE	MAE	MSE	MAE	MSE
CAN [48]	62.3	100.0	7.8	12.2	107.0	183.0	106.3	386.5
SFCN [49]	64.8	107.5	7.6	13.0	102.0	171.4	105.7	424.1
S-DCNet [50]	58.3	95.0	6.7	10.7	104.4	176.1	-	-
BL [51]	62.8	101.8	7.7	12.7	88.7	154.8	105.4	454.2
MBTTBF [52]	60.2	94.1	8.0	15.5	97.5	165.2	-	-
KDMG [53]	63.8	99.2	7.8	12.7	99.5	173.0	100.5	415.5
LSCCNN [54]	66.5	101.8	7.7	12.7	120.5	218.2	-	-
ASNet [55]	57.8	90.1	-	-	91.6	159.7	-	-
AMRNet [56]	61.5	98.3	7.0	11.0	86.6	152.2	-	-
NoiseCC [57]	61.9	99.6	7.4	11.3	85.8	150.6	96.9	534.2
DM-Count [58]	59.7	95.7	7.4	11.8	85.6	148.3	88.4	388.6
LB-Batch [59]	65.8	103.6	8.6	13.9	113.0	210.0	-	-
AutoScale [60]	65.8	112.1	8.6	13.9	104.4	174.2	94.1	388.2
GL [61]	61.3	95.4	7.3	11.7	84.3	147.5	79.3	346.1
D2CNet [62]	57.2	93.0	6.3	10.7	81.7	137.9	85.5	361.5
P2PNet [63]	52.7	85.1	6.3	9.9	85.3	154.5	77.4	362.0
SDA+DM [64]	55.0	92.7	-	-	80.7	146.3	-	-
Chfl [65]	57.5	94.3	6.9	11.0	80.3	137.6	76.8	343.0
RSI-ResNet50 [66]	54.8	89.1	6.2	9.9	81.6	153.7	-	-
DMCNet [67]	58.5	84.6	8.6	13.7	96.5	164.0	-	-
GAPNet [68]	67.1	110.4	9.8	15.2	118.5	217.2	174.1	514.7
DRMICrowd [69]	57.7	97.5	-	-	97.2	156.4	-	-
MRSNet (ours)	54.2	88.5	6.3	9.7	78.5	130.4	69.3	319.7

**Table 2:** Comparison of experimental results between TRANCOS and MTC datasets. Comparison of experimental results first (red), indicators second (blue).

Methods	TRANCOS		MTC	
	MAE	MSE	MAE	MSE
FCN-HA [70]	4.2	-	-	-
TasselNetv2 [71]	-	-	5.4	8.8
S-DCNet [50]	2.9	-	5.6	9.1
CSRNet [72]	3.6	-	9.4	14.4
RSI-ResNet [66]	2.1	2.6	3.1	4.3
MRSNet (ours)	1.7	3.0	2.6	3.7

**Table 3:** Results of ablation experiments for each module of the network. Block1 is a multi-scale feature selection module and Block2 is a dynamic sparse attention mechanism. “×” represents the absence of the module in the structure, and “√” represents the addition of the module in the structure.

Imbedding		Val Set		Test Set	
Block1	Block2	MAE↓	MSE↓	MAE↓	MSE↓
×	×	8.3	12.8	11.9	16.6
√	×	7.8	12.1	7.8	12.0
×	√	7.6	11.3	9.7	13.4
√	√	6.8	10.1	6.3	9.7

**Table 4:** Influence of different permutations and combinations of modules on the network.

Methods	Val Set		Test Set	
	MAE↓	MSE↓	MAE↓	MSE↓
PGO	8.5	12.4	9.8	14.6
POG	37.4	58.2	38.2	56.8
OPG	20.7	17.6	18.7	17.4
OGP	7.6	11.3	9.7	13.4
GPO	9.0	15.8	10.5	15.5
GOP	6.8	10.1	6.3	9.7

## Data Availability

The experimental study is supported as open data. SHHA and SHHB are available at https://www.datafountain.cn/datasets/5670 (accessed on 7 July 2024). UCF-QNRF is available at https://www.crcv.ucf.edu/data/ucf-qnrf/ (accessed on 7 July 2024). NWPU-Crowd is available at https://gjy3035.github.io/NWPU-Crowd-Sample-Code/ (accessed on 14 July 2024). TRANCOS is available at https://gram.web.uah.es/data/datasets/trancos/index.html (accessed on 14 July 2024). MTC is available at https://drive.google.com/file/d/1sNc8dzrcmC3lGifPtW_mddwcWb3YulnR/view (accessed on 15 July 2024).
